# Bee gomogenat enhances the healing process of diabetic wounds by orchestrating the connexin-pannexin gap junction proteins in streptozotocin-induced diabetic mice

**DOI:** 10.1038/s41598-023-47206-5

**Published:** 2023-11-15

**Authors:** Leila H. Sayed, Gamal Badr, Hossam El-Din M. Omar, Sary Khaleel Abd Elghaffar, Aml Sayed

**Affiliations:** 1 Zoology Department, Faculty of Science, Assiut University, Assiut, 71516 Egypt; 2 Laboratory of Immunology, Zoology Department, Faculty of Science, Assiut University, Assiut, 71516 Egypt; 3 Pathology and Clinical Pathology Department, Faculty of Veterinary Medicine, Assiut University, Assiut, 71516 Egypt; 4 School of Veterinary Medicine, Badr University, Assiut, Egypt; 5 Mallawi Specialized Hospital, 26Th of July Street, Mallawi, Minia Egypt

**Keywords:** Biochemistry, Cell biology, Immunology, Physiology

## Abstract

Delay in wound healing remains one of diabetes's worse side effects, which increases mortality. The proposed study sought to scrutinize the implications of bee gomogenat (BG) on diabetic's wound closure in a streptozotocin-(STZ)-enhanced type-1 diabetes model’s rodents. We used 3 different mice groups: group 1 non-diabetic rodents "serving as control", group 2 diabetic rodents, and group3 BG-treated diabetic rodents. We noticed that diabetic rodents experience a delayed wound closure, which emerged as a significant (*P < 0.05) decline in the deposition of collagen as compared to control non-diabetic animals. We noticed that diabetic rodents have a delayed wound closure characterized by a significant (*P < 0.05) decrease in the CD31 expression (indicator for wound angiogenesis and neovascularization) and an apparent elevation in the expression of such markers of inflammation as MCP-1 and HSP-70 as compared to control animals. Moreover, diabetic animals displayed a significant (*P < 0.05) increase in the expression of gap junction proteins Cx43 and a significant decrease in the expression of Panx3 in the wounded skin tissues when compared to the controls. Intriguingly, topical application with BG on the diabetic wounded skin tissues contributes to a significant (^#^P < 0.05) enhancing in the collagen deposition, up-regulating the level of CD31 expression and a significant (^#^P < 0.05) down-regulation in the MCP-1 and HSP-70 expressions as compared to diabetic non-treated animals. The expression's levels of Cx43 and Panx3 were significantly (^#^P < 0.05) retrieved in diabetic rodents after BG treatment. Taken together, our findings showed for the first time that BG promotes the recovering process and accelerated the closure of diabetic related wounds.

## Introduction

The recovering of wound belongs to a natural process that encompasses several stages: inflammation, granulation tissue formation, new structures production and tissue remodeling^1^. Diabetes mellitus (DM) is frequently associated with delayed or impaired wound healing, which is manifested by dwindled collagen production and abnormal angiogenesis^2^. The impaired recovering of wounds is regarded as one of the major diabetes complications that occur due to the release of free radicals^3^ which destruction several constituents in cell, including lipids, proteins and DNA. Additionally, impaired wound recovery occurs in diabetes wounds due to elevated apoptosis, diminished vascular recovery, an abnormal inflammatory response, and slower cell turnover^4,5^. Due to hyperglycemia, the recovering wound process in diabetic patients deteriorates, resulting in chronic wounds and limb amputation^6^. During healing of diabetic related wounds, numerous dysregulated cellular functions are involved including decreased T-cell immunity and phagocytosis, increased bactericidal activity, and epidermal cell dysfunction^7^. Angiogenesis or neo-vascularization to supply shattered tissues, is an essential process for wound healing, and its disturbance contributes to the emergence of non-healing skin ulcers related to diabetes^8^. Wound recovering of the skin is dependent on a tightly controlled dynamic interplay between the extracellular matrix and cells^9^, which necessitates intercellular communication that mediated throughout skin's gap junctions. Direct intracellular and cell-extracellular communications are required for skin homeostasis to be maintained^10,11^. Gap junction proteins consist of the pannexin (Panx) and connexin (Cx) and protein families^12^. Gap junction proteins serve as channels that permit the passage of different substances suchlike second messengers, morphogens, ions, and metabolites between adjacent cells^10,11^. The pannexin family includes Panx3, which is only found in the epidermis. Unlike Panx1 or Cx43, which are found in both the dermis and the epidermis^13,14^. Panx3 loss causes severe skeletal abnormalities in mice^15,16^. Panx3 is a channel that has diverse roles depending on where it is located in the cell. Panx3 activates the PI3K/AKT pathway by releasing ATP from intracellular location into the extracellular space at the plasma membrane. Such AKT phosphorylation triggers the endoplasmic reticulum (ER)-related Panx3 to elevate intracellular concentrations of Ca2 + , that promotes differentiation by up-regulating the calmodulin-calcineurin-NFAT pathway^16,17^. Panx3 deficiency impairs wound recovery by preventing epithelial–mesenchymal transition, keratinocyte migration, and collagen skin remodelling^18^. The epidermis contains the protein Cx43, which has been shown to affect keratinocyte migration, proliferation, and differentiation^19^. Previous studies demonstrated that Cx43 is increased in chronic lower limb wounds or ulcers of diabetes, indicating that down-regulation is necessary for wound normal recovering and that dysregulation of such "channel" protein is associated with delayed chronic wound closure^20,21^.

Bee gomogenat (BG) is a nutrient-rich substance found in trutnevy larvae that include proteins, minerals (phosphorus, potassium, calcium, magnesium, zinc, iron), vitamins; β-carotene, A, B, E and D, different enzymes, steroid hormones and amino acids. We recently demonstrated the effects of BG on the structure of the lymphoid's organs and the immune responses in STZ-enhanced type-I diabetic (T1D) mice’s' models^22^. However, in the proposed study we extend our work to evaluate the therapeutic possibility of topically applicated BG on the recovering process of diabetic related wounds, that is regarded as among the most significant diabetes consequences.

## Results

### Biochemical evaluation of the body weight and blood glucose concentrations in a diabetic mouse model

During the experiment, we monitored variations in the glucose concentrations as well as the body weights among three studied groups of rodents. In comparison to the control mice, STZ induced significant hyperglycemia depicted by a significant rise in glucose concentrations, but a decline in body weight, respectively (n = 20, *P < 0.05), (Tables 1 and 2). Interestingly, BG treatment exhibited an ameliorative significant (*P < 0.05) effect on the body weight and blood glucose concentration of diabetic mice compared to untreated diabetic mice.

**Table 1:** Impact of BG treatment on the blood concentrations of glucose in the three animal groups.

Blood glucose (mg/dl)			
Days post-wounding	Animal groups		
	Cont	Diab	Diab. + BG
Day 0	129 ± 4.62	301.3 ± 6.96*	275 ± 14.43^+^
Day 3	151.7 ± 2.40	314.7 ± 2.91*	291.7 ± 8.33^+#^
Day 6	155 ± 15.53	333.3 ± 12.02*	285 ± 13.23^+#^
Day 9	133 ± 6.93	353.3 ± 8.82*	281.7 ± 16.41^+#^
Day 12	152.7 ± 10.04	408.3 ± 6.01*	260.7 ± 9.24^+#^
Day 15	132.7 ± 11.26	456.7 ± 23.33*	246.7 ± 8.82^+#^

We measured blood glucose concentrations at the designated time points after injury. The pooled data for 20 mice from each group are presented as mean ± SEM.

**Table 2:** Effect of diabetes induction and BG treatment on the body weights in the three animal groups.

Body weight (g)			
Days post-wounding	Animal groups		
	Cont	Diab	Diab. + BG
Day 0	27.97 ± 1.613	28.67 ± 2.40	28.10 ± 1.07
Day 3	28.67 ± 1.764	28.07 ± 1.09	29.17 ± 1.17
Day 6	30.83 ± 0.726	27.87 ± 1.55	30.47 ± 0.29
Day 9	31.33 ± 0.8819	26.93 ± 0.5812*	30.63 ± 1.304^#^
Day 12	32.67 ± 1.453	25.50 ± 0.7638*	32.93 ± 0.9684^#^
Day 15	33.33 ± 0.8819	25.17 ± 0.1667*	33.67 ± 0.8819^#^

The body weights were measured at the designated time points after injury. The pooled data for 20 rodents from each group are presented as mean ± SEM.

### BG hastens wound closure in mice suffering diabetes.

**Figure 1:**
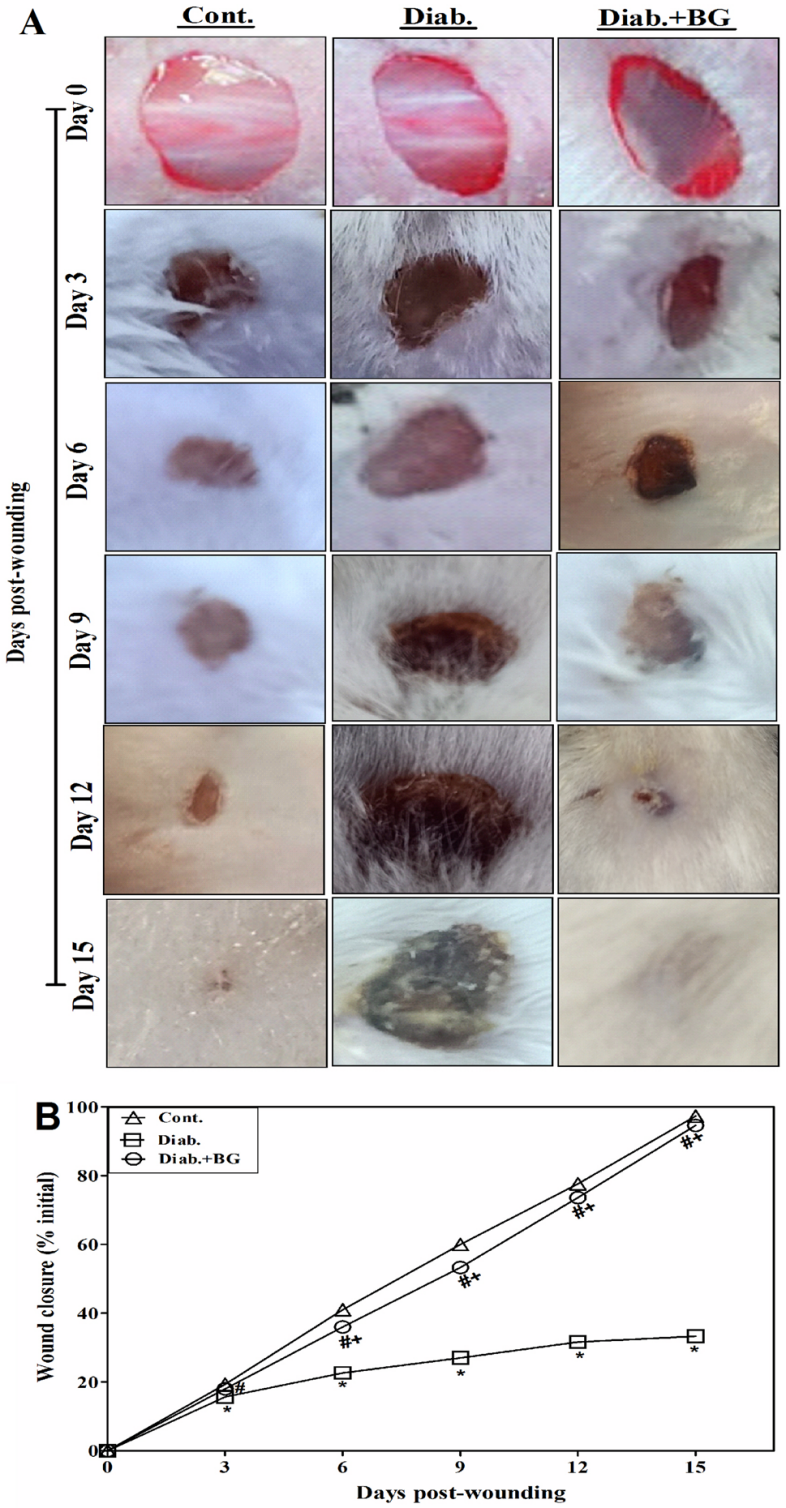
Effect of BG treatment on wound closure. (**A**) The wound areas were photographed at the designated times. The images from day zero were taken instantaneously following the injury. Each group's illustrative data are drawn from 20 individual mice that are shown. (**B**) The data that was gathered for variations in the percentage of wound closure from 20 individual animals in each group is displayed.

Considering photographs taken on days 0, 3, 6, 9, 12, and 15, we assessed the wound closure percentage from the moment of wounding up to the 15th day. Data indicated that all three groups of rodents at days 0 and 3 after-wounding had wound sites with similar sizes and morphology. Whereas, on days 6, 9, 12 and 15 after wounding, both BG-treated diabetic mice and controls exhibited comparable percentages of wound closure (Fig. 1A). Data collected from 20 mice in each group proved that topical application with BG on such diabetic related wounds significantly hastened the recovering mechanism of diabetic related wounds than untreated diabetic ones which showed delayed and impaired wound closure (Fig. 1B).

### BG treatment repairs the histological alterations in skin wounded tissues in diabetic rodents.

**Figure 2:**
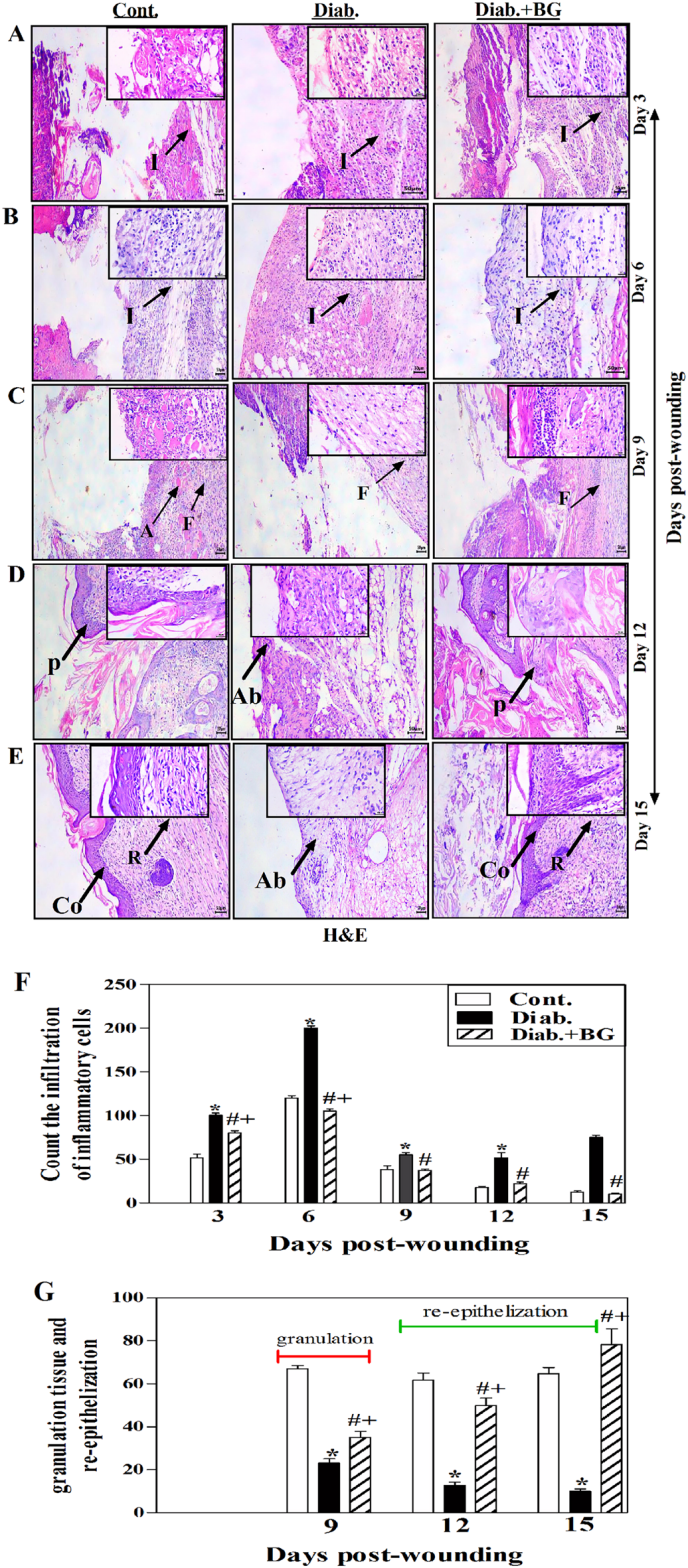
Microscopic examination of wound tissues, staining with Hematoxylin and Eosin (H&E). H&E staining of (**A**) Day-3; (**B**) Day-6; (**C**) Day-9; (**D**) Day-12 and (**E**) Day-15 wounds; and (**F**) quantification of the infiltrated inflammatory cells and (**G**) quantification of the granulation tissue and re-epithelization (× 100). H&E for the examination of histopathological changes in skin wounded tissues from days 3 to15 day post-wounding. Because two wounds were made on the back of each mouse, hence n = 6 wounds from 3 animals per each time point from each group. I: infiltration of inflammatory cells, A: angiogenesis, F: fibrogenesis, P: partial re-epithelization, Ab: absence re-epithelization, Co: complete re-epithelization, R: remodeling. The inset rectangle shows higher magnification of the stained infiltration of inflammatory cells, angiogenesis, fibrogensis, partial re-epithelization, absence re-epithelization, complete re-epithelization and remodeling (400$\times$).

The histopathological changes of wounded skin tissues from 3 to 15 days after-wounding were monitored using H&E staining method and the results are shown in Fig. 2A–E. Diabetic rodents on 3 and 6 days after-wounding experienced an impressive rise in the infiltration of inflammatory cells comparing to the controls and exhibited as a mild infiltration of inflammatory cells. However, diabetic rodents treated with BG displayed moderate infiltration of inflammatory cells compared to untreated diabetic rodents (Fig. 2A,B). Additionally, diabetic rodents on day 9 after-wounding displayed a decrease in the granulation tissue represented by fibrogensis as compared to control rodents which exhibit an increase in the granulation tissue that was noticed in the form of angiogenesis and fibrogensis. Interestingly, topical application with BG on the diabetic wounded skin tissues exhibited increase fibrogensis compared to untreated diabetic rodents (Fig. 2C). At 12 days post-wounding, diabetic rodents showed absence of re-epithelization (impaired or delayed recovering process) compared control group which exhibit partial re-epithelization followed by thickening of epidermis (cornified stratified squamous epithelium). Treatment of diabetic mice with BG indicated partial re-epithelization compared to untreated diabetic rodents (Fig. 2D). Finally, at 15 days after-wounding diabetic rodents displayed absence of re-epithelization as compared to control group which display complete re-epithelization and remodeling of collagen fibers. Interestingly, treatment of diabetic rodents with BG displayed complete re-epithelization followed by thickening of epidermis with formation of rete-redges and remodeling of collagen fibers compared to untreated diabetic mice (Fig. 2E). We then quantified the infiltration of inflammatory cells (neutrophils and macrophage) using image-J and pooled data from 3 rodents at each time point in each group is shown. Diabetic rodents exhibited a significant (*P < 0.05) and sustainable increase in the infiltration levels of inflammatory cells compared to non-diabetic controls. Diabetic mice treated with BG significantly decreased the infiltration levels of inflammatory cells nearly to those seen in controls (Fig. 2F). Additionally, we quantified the granulation tissue and re-epithelization at each time point using image-J software. Collected data from three different rodents from each group illustrating that diabetic mice exhibited a marked decreased in the granulation tissue (seen only at day 9 post-wounding) and re-epithelization (from day 12 to day 15 post-wounding) than observed in non-diabetic controls. Diabetic mice treated with BG significantly retrieved the granulation tissue and re-epithelization close to that seen in control animals (Fig. 2G).

### BG treatment increases collagen production in skin wounded tissues of diabetic animal model.

**Figure 3:**
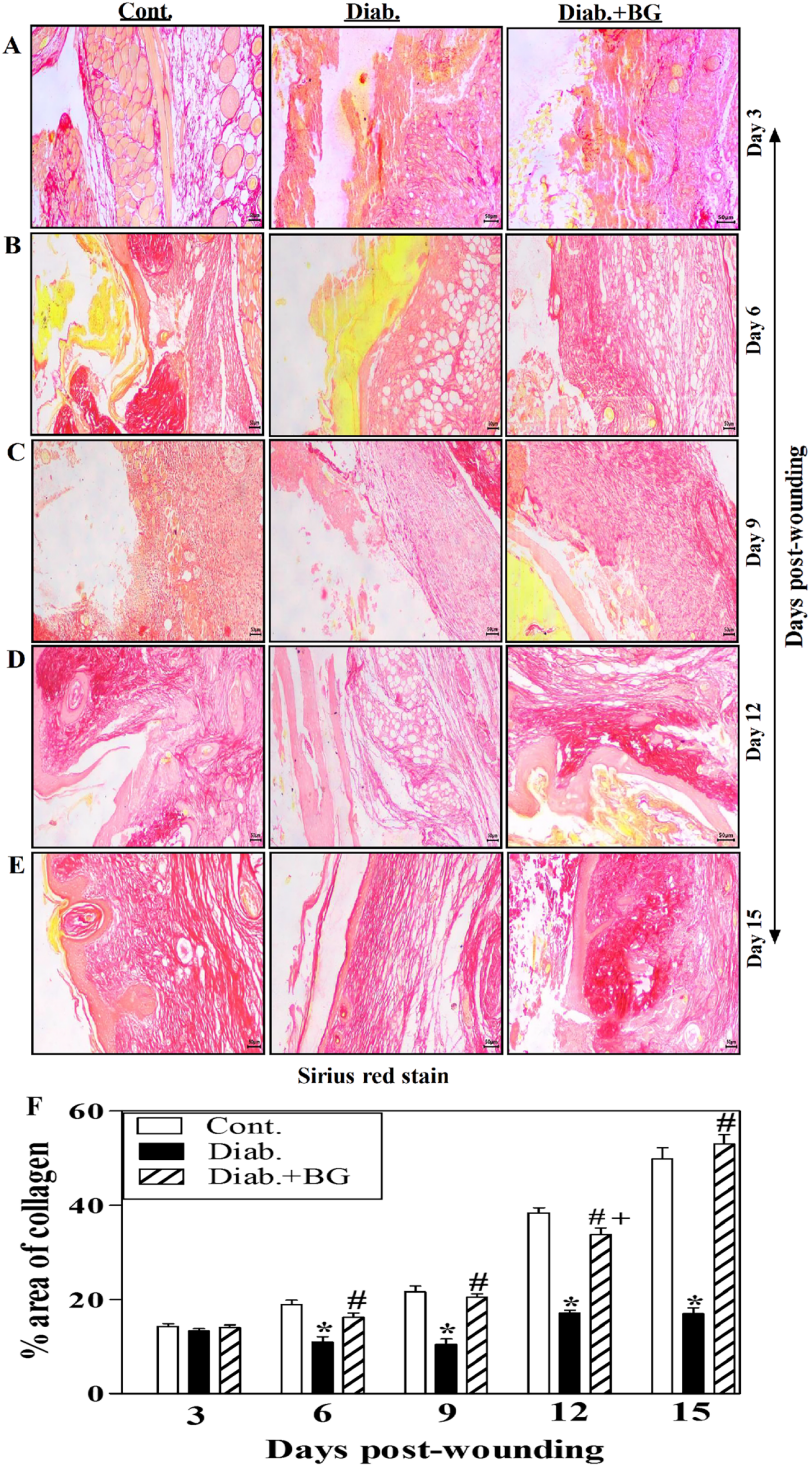
Influence of BG on collagen formation in wounded skin sections stained with Sirius red. Sirius red staining of (**A**) Day-3; (**B**) Day-6; (**C**) Day-9; (**D**) Day-12; (**E**) Day-15 wounds; and (**F**) quantitative deposition of collagen (100x) (n = 6 wounds from 3 animals/time point/each group).

Figure 3A–E demonstrates one illustrative experiment for tissue sections from BG-treated diabetic, diabetic and control groups to illustrate the deposition of collagen as stained by Sirius red stain. The three animal groups had a similar collagen deposition on day 3 following wounds (Fig. 3A). In contrast to non-diabetic control mice, diabetes rodents showed a substantial reduction in collagen deposition from days 6 to 15 following wounds. Diabetic rodents treated with BG clearly enhanced the process of collagen deposition close to that observed in controls (Fig. 3B–E). At each time point, we quantify the deposition of collagen using image-J and collected data from 3 rodents in each group, in which diabetic rodents displayed a marked decline in the collagen deposition compared to non-diabetic controls. Treatment with BG significantly improved collagen deposition in diabetic rodents to levels similar to those seen in the controls (Fig. 3F).

### BG treatment improves HSP-70 expression in skin wounded tissues of diabetic animal model

**Figure 4:**
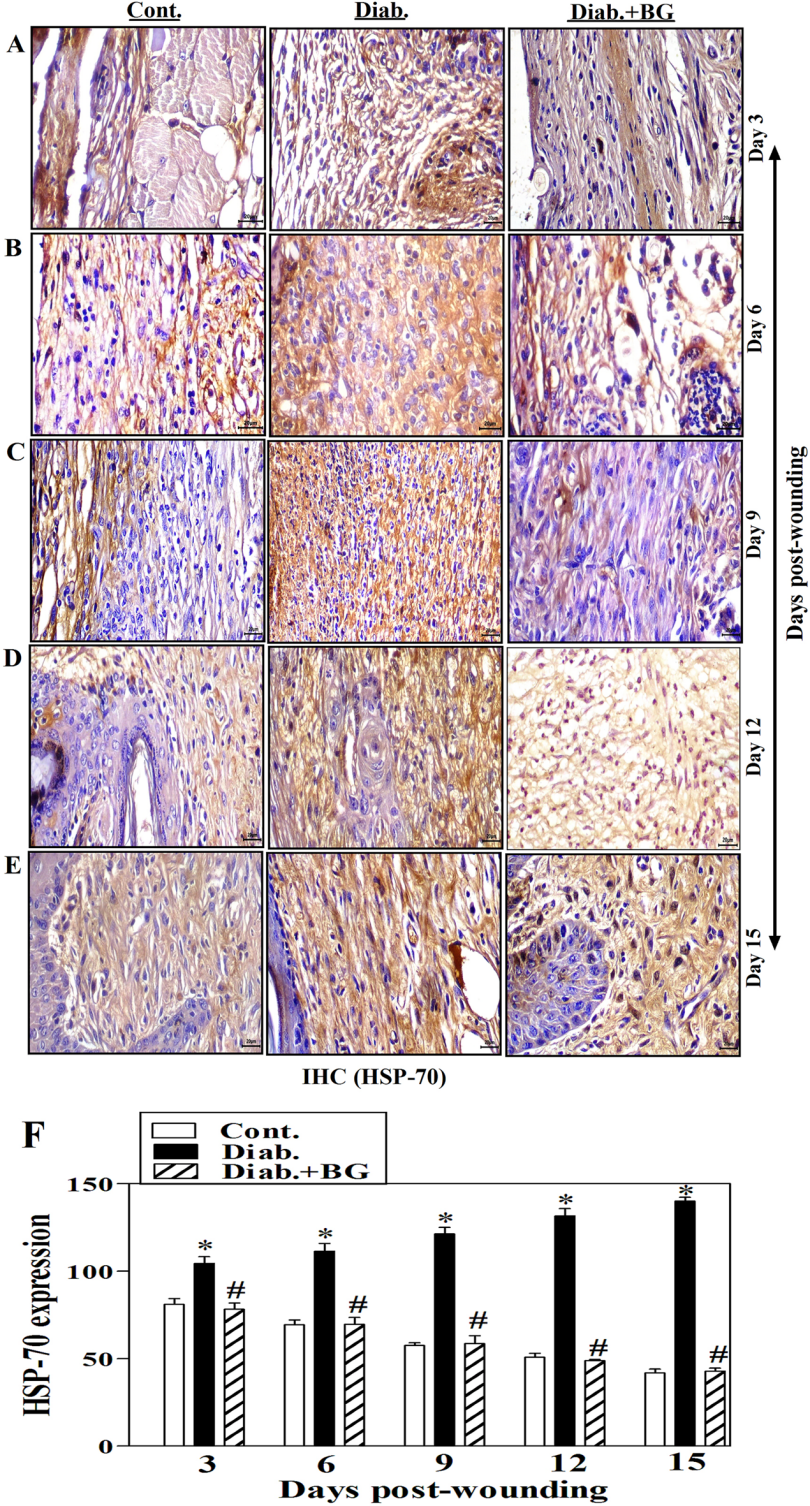
Impact of BG treatment on the expression of HSP-70 in wounded skin tissues diabetic mice. HSP-70 staining of (**A**) Day-3; (**B**) Day-6; (**C**) Day-9; (**D**) Day-12; (**E**) Day-15 wounds; and (**F**) quantification of HSP-70 expression (400$\times$) (n = 6 wounds from 3 animals/time point/each group).

HSP-70 is a stress-inducible protein, and its expression is up-regulated in various states of physiological and environmental stresses, such as inflammation, infections, and diabetes, and hence it is considered as a marker of inflammation. Figure 4A–E displays one illustrative experiment for tissue sections BG-treated diabetic, diabetic and control groups to demonstrate the Heat shock protein-70 (HSP-70) expression as stained by immunohistochemistry (IHC) technique. Compared to control non-diabetic mice, diabetes rodents showed noticeably higher levels of HSP-70 expression from days 3 to 15 following wounds. What's more, diabetic animal treatment with BG clearly reduced HSP-70 expression to practically that of control animal levels (Fig. 4A–E). At each time point and by applying image-J software, we quantified the expression of HSP-70 and collected data from three different rodents found in each group, establishing that diabetic mice exhibited a marked elevation in the expression of HSP-70 than observed in non-diabetic controls. Diabetic mice treated with BG significantly retrieved the expression of HSP-70 close to that seen in controls (Fig. 4F).

### BG treatment enhances MCP-1 expression in skin wounded tissues of diabetic animal model

**Figure 5:**
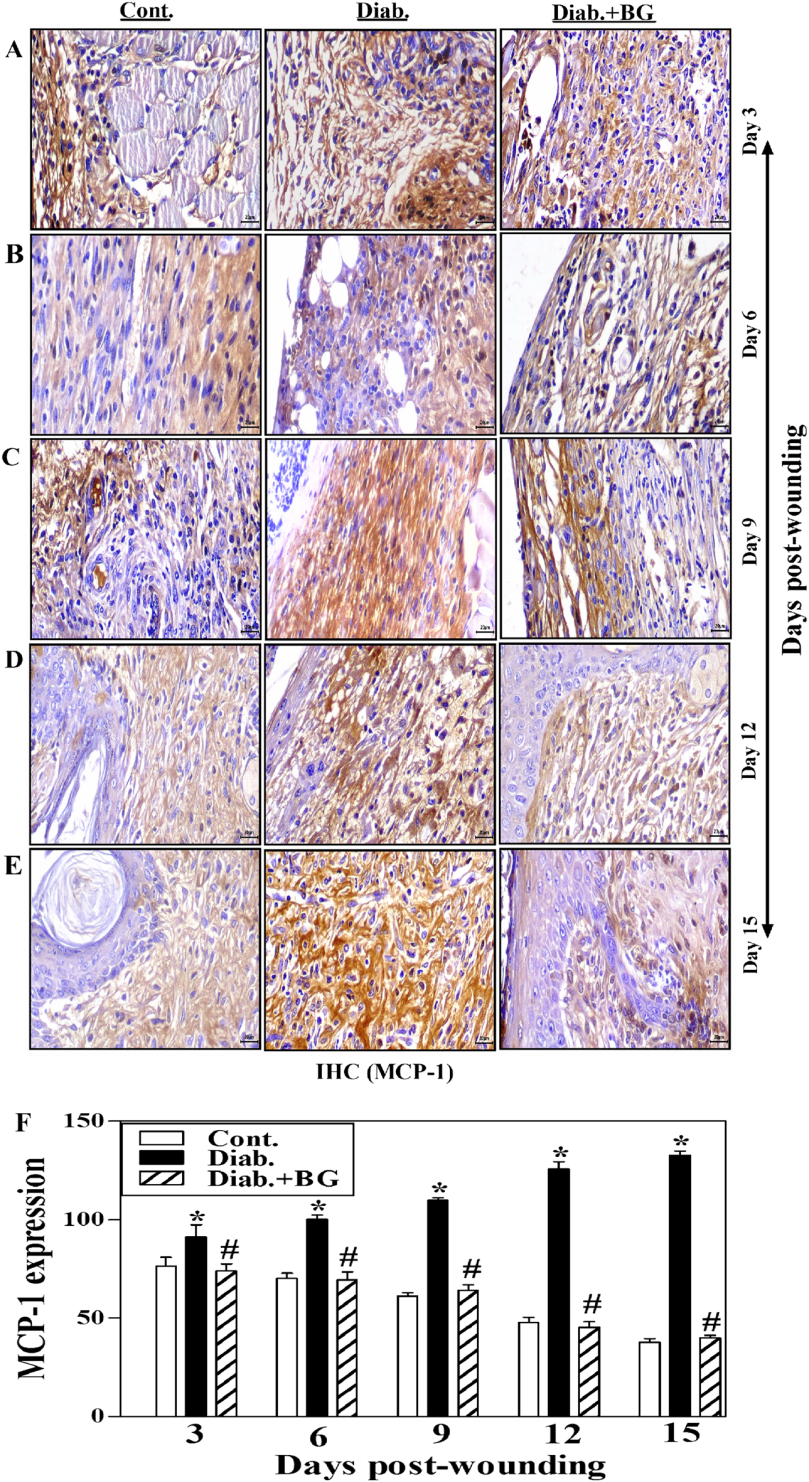
Topical application with BG boosts the expression of MCP-1 in wounded skin tissues of diabetic mice. MCP-1 staining of (**A**) Day-3; (**B**) Day-6; (**C**) Day-9; (**D**) Day-12; (**E**) Day-15 wounds; and (**F**) quantification of MCP-1 expression (400$\times$) (n = 6 wounds from 3 animals/time point/each group).

Figure 5A–E depicts one illustrative experiment for tissue sections from BG-treated diabetic, diabetic and control groups were employed to show the expression of Monocyte Chemoattractant Protein-1 (MCP-1) as detected by IHC method. From days 3 to 15 post-wounding, diabetic mice were shown to have significantly higher levels of MCP-1 expression than non-diabetic control rodents. Following BG therapy, diabetic rodents' reduced MCP-1 expression's levels were very close to those seen in the control group (Fig. 5A–E). We quantified MCP-1 expression using image-J and collected data from three mice from each group at each designated time revealed that diabetic animals had a higher significant level of MCP-1 expression than non-diabetic controls. Most intriguingly, after treatment with BG, diabetic mice's MCP-expression significantly decreased, approaching that of control rodents (Fig. 5F).

### BG treatment restores the expression of CD31 in skin wounded tissues of diabetic mice model.

**Figure 6:**
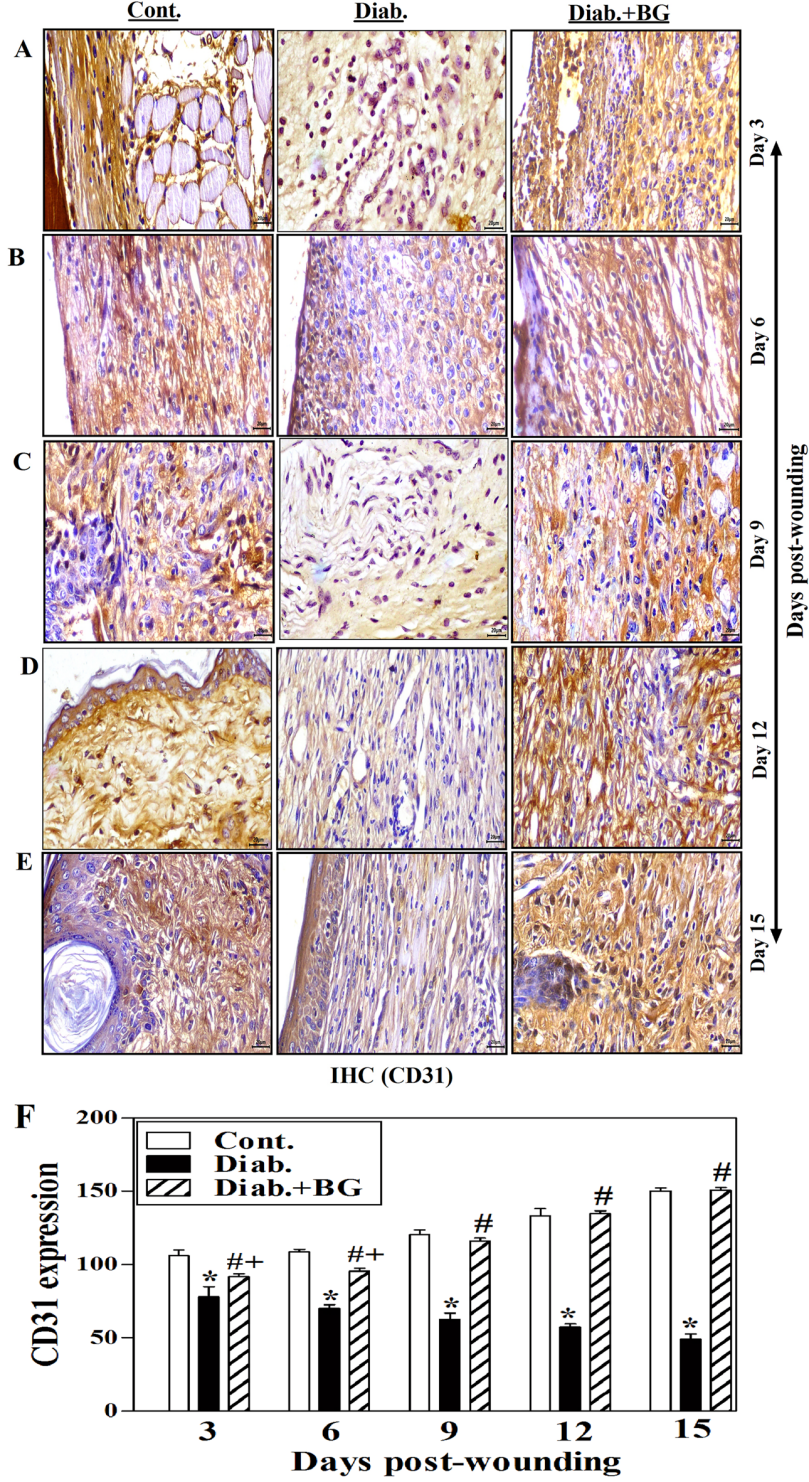
CD31 staining of (**A**) Day-3; (**B**) Day-6; (**C**) Day-9; (**D**) Day-12; (**E**) Day-15 post-wounding; and (**F**) quantitative angiogenesis expression (400$\times$) and scale bar = 20 µm; (n = 6 wounds from 3 animals/time point each group).

Figure 6A–E demonstrates one illustrative experiment for tissue sections from BG-treated diabetic, diabetic and control groups to illustrate the expression of CD31 as detected by IHC technique. Compared to the non-diabetic control rodents, diabetic mice demonstrated a clear drop in CD31 expression from days 3 to 15 following wounds. As diabetic rodents were treated with BG, there was an obvious increase CD31 expression close to those observed in control non-diabetic rodents (Fig. 6A–E). At each time point, quantification CD31 expression was analyzed by image-J and pooled data from three mice in each group. The results showed that diabetic mice had significantly lower CD31 expression than non-diabetic controls. Nevertheless, topical application with BG on the diabetic wounded skin tissues significantly retrieved the CD31 expression, that were closely comparable to those in the non-diabetic control animal (Fig. 6F).

### BG restores the expression of Cx43 in skin wounded tissues of diabetic mice model.

**Figure 7:**
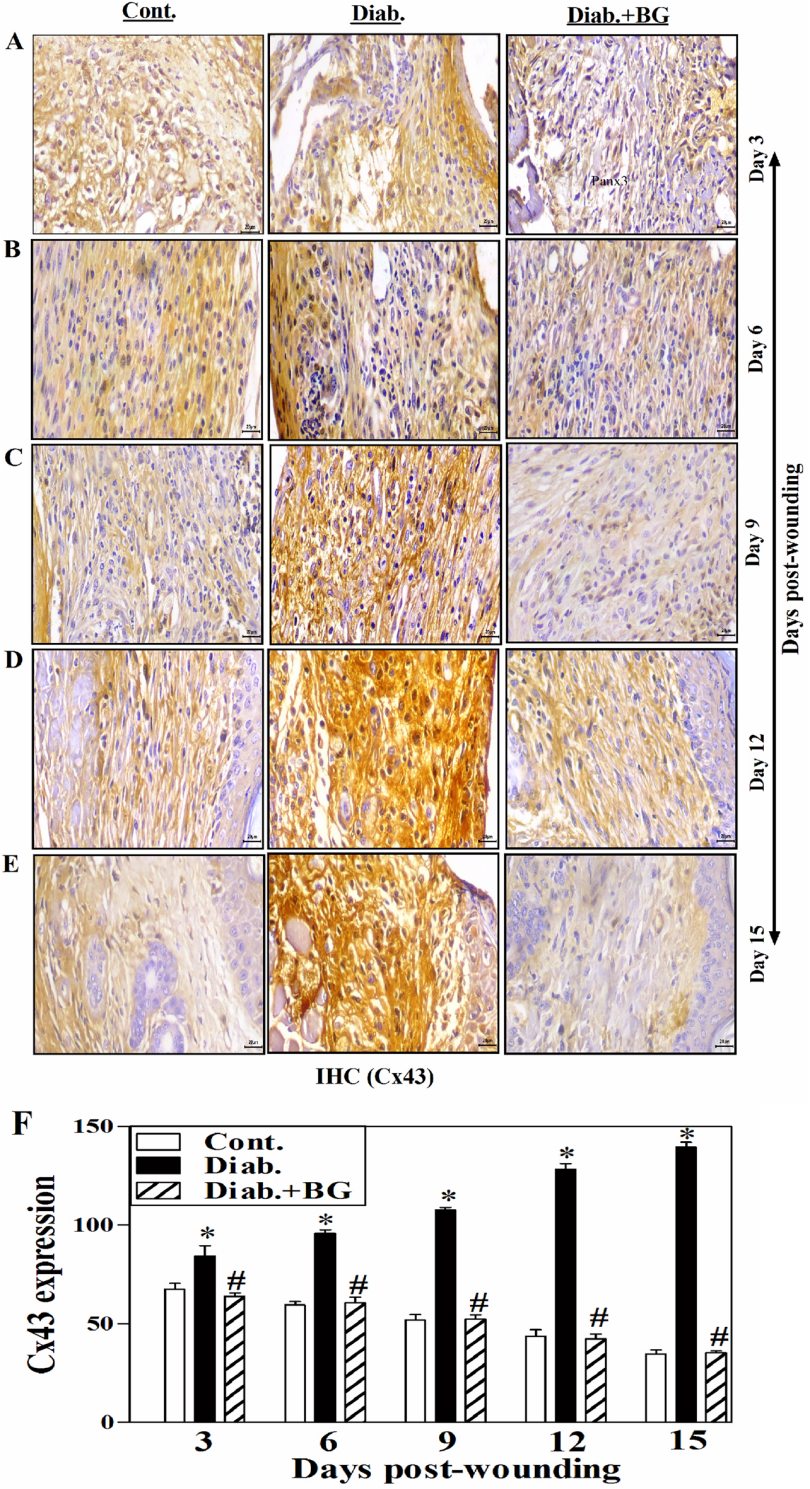
Influence of BG treatment on the expression of Cx43 in wounded skin tissues of diabetic mice. Cx43 staining of (**A**) Day-3; (**B**) Day-6; (**C**) Day-9; (**D**) Day-12; (**E**) Day-15 wounds; and (**F**) quantification of Cx43 expression (400$\times$) (n = 6 wounds from 3 animals/time point/each group).

Figure 7A–E displays one illustrative experiment for tissue sections from BG-treated diabetic, diabetic, control groups to show the expression of Cx43 as detected by IHC technique. From days 3 to 15 after-wounding, it was found that diabetic mice showed a substantial rise in the "Cx43" expression compared to non-diabetic control group. As compared the non-diabetic controls, diabetic mice showed a noticeably higher expression of Cx43 from days 3 to 15 following wounds (Fig. 7A–E). Three rodents from each group at each designated time point were utilized for the Cx43 expression quantification when applying image J. software. The findings suggested that diabetic mice had a significant greater amount of Cx43 expression than non-diabetic control rodents. Expression of Cx43 was declined significantly in diabetic mice given BG, just like it was in control mice (Fig. 7F).

### BG treatment increases the expression of Panx3 in skin wounded tissues of diabetic mice

**Figure 8:**
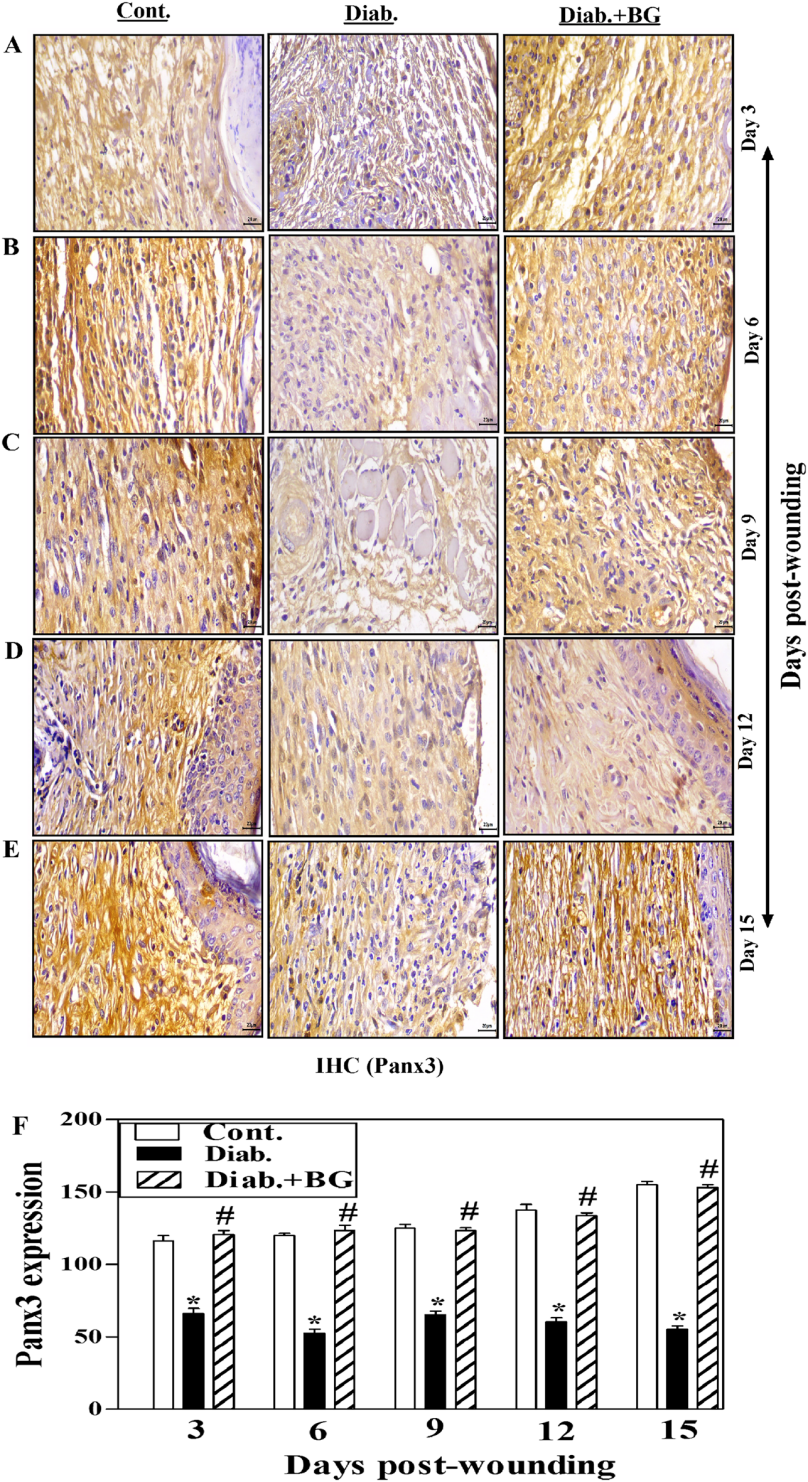
Influence of BG treatment on the expression of Panx3 in wounded skin tissues of diabetic mice. Panx3 staining of (**A**) Day-3; (**B**) Day-6; (**C**) Day-9; (**D**) Day-12; (**E**) Day-15 wounds; and (**F**) quantification of Panx3 expression (400$\times$) (n = 6 wounds from 3 animals/time point/each group).

One illustrative experiment for tissue sections from the BG-treated diabetes, diabetic and control groups is shown in Fig. 8A–E to clarify the expression of Panx3 as detected by IHC method. Compared to the non-diabetic controls, diabetic rodents revealed an apparent drop in Panx3 expression from days 3 to 15 after-wounding. When diabetic mice were treated with BG, there was a noticeable rise in the expression of Panx3 nearly to those found in non-diabetic controls (Fig. 8A–E). Quantification using Image-J to examine Panx3 expression and collected data from 3 mice from each group at each designated time point showed that diabetic rodents exhibited significantly decreased Panx3 expression compared to control non-diabetic rodents. Nevertheless, topical application with BG on the diabetic wounded skin tissues significantly raised the Panx3 expression just like those in non-diabetic controls (Fig. 8F).

## Discussion

Continuous diabetes-associated complications cause immune system exhaustion and infection. Diabetes can result in impaired blood flow to the feet, which raises the chance of developing ulcers when the skin is injured and thus delays the process of healing^23^. Our findings disclosed that the delayed wound recovering in diabetic rodents was connected to a considerable rise in blood glucose concentrations and a notable drop in body weight, yet these influences are partially retrieved by BG therapy. Similarly, selenium nanoparticle therapy raised body weight and lowered blood glucose concentrations in diabetic mice^24^. The wounds in the current study were examined, and it was discovered that although DM tardiness the skin recovering process, topical administration of BG after wounding speeds wound closure in diabetic mice. Likewise, whey protein (WP) supplementation hastened diabetic wound closure^25^. In this work, we noticed that defective wound's recovering in the diabetic rodents was correlated with an increased in the infiltration of inflammatory cells, declined in the granulation tissue, absence of re-epithelization and reduced deposition of collagen fibers, consistent with the results of previous studies^26,27^. Previous study revealed that the reduced deposition of collagen in the acute wounds of T1D patients is likely owing to the decrease in the proliferation of fibroblast^28^. BG treatment reduced the inflammatory cells' infiltration in diabetic rodents, increased granulation tissues and deposition of collagen fibers, these lead to complete re-epithelization and accelerated wound recovering process. Therefore, we suggest that BG could be a new effective strategy for improving the recovering process in diabetes. Although the data are clear with H&E staining, there are difficulties in clarifying the infiltration of leukocytes with H&E staining in this study. Accordingly, in our further study we will use a panel of specific antibodies such as Ly6G and F4/80 to monitor and investigate the infiltration of specific leukocytes in the wounded tissues.

HSP-70 is a stress protein family that can be found in a wide range of organisms. HSP-70 modulates intracellular proteins homeostasis and inhibits the toxic aggregates from formation, causing inflammation or even cell death^29^. MCP-1 is a strong chemoattractant that is expressed early after-wounding and is necessary for monocyte/macrophage response and appropriate recovering^30^. By controlling angiogenesis in endothelial cells, HSP-70 and MCP-1 can accelerate the recovering of wounds^31,32^. Cellular stress causes elevation in HSP-70 expression that in turn effectively protects the islets of β cell isolated from rat and human against damaging mediators^33^. In line with the findings of the prior study, the current investigation found that diabetic rodents expressed higher concentrations of HSP-70 and MCP-1^34^. Regarding BG-treated diabetic rodents, HSP-70 and MCP-1 expressions were retrieved in wounded skin tissues, thereby we suggest that BG is promising a novel strategy to decrease inflammation and hasten wound healing. CD31 is a biomarker for blood vessels that plays a key function in wound recovering^35^. Hyperglycemia causes damage in blood vessels, nerves, and other organ in the body^36,37^. In diabetics, decreased peripheral blood supply and local neo-vascularization cause delayed or repair-resistant wounds^38^. It has been shown that downregulation of CD-31 expression is associated with delayed wound recovering^39^. We observed that diabetic mice had lower CD31 expression in our study, and this is consistent with other previous work^40^. Diabetic rodents treated with BG retrieved the expression's levels of CD31. Our results clearly indicate that BG effectively stimulates active angiogenesis, which improves the disturbed recovering process. Therefore, it is considered as a new strategy for improving wound closure. Gap junctions are crucial regulators of tissue homeostasis and the various processes that lead to the restoration of this critical balance, which are triggered by damage like wound healing and tissue repairing, angiogenesis, and carcinogenesis^41^. In diabetic mice with damaged skin tissues, the ongoing work's findings disclosed that the expression of Cx43 rose while the expression of Panx3 dropped. These results agree with those of a prior study^20^. In addition, abnormal Cx43 expression in the edges of wound, keratinocytes were discovered to represent the root of impaired wound recovering in diabetic mice^42^. Furthermore, deceased fibroblast migration is linked to up-regulation of the Cx43 protein in fibroblasts of diabetic foot ulcers, which impairs wound recovering^43^. Further on, inadequate re-epithelialization, a diminished inflammatory response, and impaired collagen remodeling were all seen in Panx3-knockout rodents, which also showed a substantial delay in wound healing^18^. Furthermore, topical application of BG to diabetic skin tissues that had been injured retrieved Cx43 and Panx3 expression's levels that seen in the controls. Novel approaches should be used to reduce diabetes complications because they can even pose a life-threatening. Since, BG enhances the deposition of collagen, the over-expression of CD31, down-regulates MCP-1 and HSP-70 expression, restores Cx43 and Panx3 expression's levels without causing any negative side effects, suggesting that it would be a novel way to deal with the serious repercussions of diabetes (impaired wound healing).

## Conclusions

There is not any research available on the biological effects of BG on the wound recovering process of diabetic related wounds. Therefore, administering BG to diabetic mice speeds wound recovering by increasing collagen deposition, increasing CD31 expression, down-regulating the pro-inflammatory markers MCP-1 and HSP-70 expression, and restoring Cx43 and Panx3 expression levels. Our results displayed, for the unprecedented time, that the BG treatment of diabetic related wounds improves the recovering process and accelerates the wound closure considered among the major health consequences of diabetes in diabetic mice model.

## Materials and methods

### Preparation of bee gomogenat

Bee gomogenat (BG) (bee milk) was bought from Etman colonies in Tanta, Egypt, which produces honey bee goods. Using GC–MS, we recently demonstrated the active chemical components of BG including proteins, minerals (phosphorus, potassium, calcium, magnesium, zinc, iron), vitamins; β-carotene, A, B, E and D, different enzymes, steroid hormones, and amino acids^22^.

### Chemicals

STZ was obtained from Sigma Chemicals Co. in St. Louis, Missouri, USA. The STZ was newly prepared for usage immediately (within 5 min) by dissolving in cold 0.01 M citrate buffer (pH 4.50). Sirius Red stain was purchased from Biognost (C.I. 35780) and H&E stain were obtained from Sigma Aldrich (h3136) and (e4382) respectively.

### Experimental design and doses

Sixty mature male BALB/C mice were purchased from Theodor Bilharz Institute in Cairo, Egypt, weighing between 25 and 30 g. The University of Assiut Faculty of Medicine's Ethics Committee authorized all animal studies, which were carried out in compliance with institutional animal care policies and international animal care standards (Council of the European Communities, 1986). As previously mentioned, we lessen animal suffering and maintain a low animal population^44^. Following a week of acclimatization, the mice were divided into three groups containing twenty rodents each: diabetic treated with BG (diab. + BG), diabetic (diab.) and controls (cont.). Intraperitoneal (i.p.) injections of STZ (60 mg per kilogram body weight) in a buffer of 0.01 Molar citrate (pH 4.5) for 5 consecutive days^45,46^ were used to cause diabetes in mice in groups 2 and 3. All the mice were fasted for 20 h prior to diabetes induction. The vehicle only was administered into the control group of mice (n = 20) (0.01 Molar citrate buffer, pH 4.5). Mice were categorized as diabetic when their glycemia surpassed 220 mg/dl.

### Preparation of an excisional wound and a macroscopic evaluation

The rodents were injured in accordance with our earlier reported methodology for two weeks following the introduction of diabetes^46^. Briefly, the mice were anesthetized with a single i.p. injection of ketamine (80 mg/kg body weight) and xylazine (10 mg/kg body weight). The back of each mouse was shaved and cleaned with 70% ethanol. Two wounds (8 mm in diameter, 3–4 mm apart) were made on the back of each mouse by excising the skin and underlying panniculus carnosus. Then, we divided the rodents into three main groups (20 mice/group): BG-treated diabetic group (group 3), diabetic group (group 2), non-diabetic control group (group 1). Groups 1 and 2 received 15 days (once a day) of topical treatment using 50 μl of distilled water per wounded region. In our laboratory, we determined the BG dose according to the previously determined LD50. A sterile cotton bud was used to paint BG onto the entire wound surface (once a day for 15 consecutive days). Finally, at the designated time points, we examined the diameter of the wounded tissues to assess the degree of wound closure.

### Blood analysis

The Rightest® Blood Glucose Monitoring System GM600 (Bionime) was implemented to monitor the blood sugar concentration. All the mice were fasted prior measuring the blood glucose levels.

### Histopathological and immunohistochemistry study

On days 3, 6, 9, 12, and 15 after injury, a sample was obtained from each group of rodents for histological examination. As previously mentioned, and by using formal alcohol, the skin specimens were quickly fixed until processed^47^. Each sample was then dehydrated, embedded and thin (3 μm) sections cut. For histopathological evaluation, sections underwent staining with Sirius red and H&E. For IHC tissue sections were prepared accordingly^48^. We used the primary antibodies listed below to stain the skin sections: anti-Cx43 and anti-Panx3 (1:200 with PBS) from Thermo Fisher Scientific Invitrogen; anti-HSP-70 (1:50 with PBS), anti-MCP-1, and anti-CD31 (1:200 with PBS) from eBioscience.

### Statistical analysis

The statistical analysis (SEM) was carried out using GraphPad Prism Version 5 for normally distributed data, represented as the mean ± SE "standard error of the mean". A one-way ANOVA and Tukey's post hoc test were used to see whether there were significant variations among the three groups or not. Differences were expressed as significant statistically at *P less than 0.05, diab. versus cont., ^+^*P* less than 0.05, diab. + BG versus cont., ^#^*P* < 0.05, diab. + BG versus diab. group.

### Ethical approval

The Research Ethical Committee of the Faculty of Science, Assiut University, Assiut, Egypt, authorized the experimental design and animal handling protocols. In conformity with the applicable ARRIVE rules and guidelines, all experimental processes were conducted. Additionally, all techniques were carried out in complying with the pertinent rules and regulations.

## Data Availability

The published manuscript includes all generated data. Contact the corresponding author, Gamal Badr, at badr73@yahoo.com and gamal.badr@aun.edu.eg, for any additional information.
