# Correction: Bee gomogenat enhances the healing process of diabetic wounds by orchestrating the connexin-pannexin gap junction proteins in streptozotocin-induced diabetic mice

**DOI:** 10.1038/s41598-025-11215-3

**Published:** 2025-07-22

**Authors:** Leila H. Sayed, Gamal Badr, Hossam El‑Din M. Omar, Sary Khaleel Abd Elghafar, Aml Sayed

**Affiliations:** 1 Zoology Department, Faculty of Science, Assiut University, Assiut, 71516 Egypt; 2 Laboratory of Immunology, Zoology Department, Faculty of Science, Assiut University, Assiut, 71516 Egypt; 3 Pathology and Clinical Pathology Department, Faculty of Veterinary Medicine, Assiut University, Assiut, 71516 Egypt; 4 School of Veterinary Medicine, Badr University, Assiut, Egypt; 5 Mallawi Specialized Hospital, 26Th of July Street, Mallawi, Minia Egypt

Correction to: *Scientific Reports* 10.1038/s41598-023-47206-5, published online 15 November 2023

The original version of the Article contained errors in Figures 1, 4, 6 and 7. Duplication occurred during the figure assembly in the third row of images in all figures. Specifically, in Figure 1, where Day 3 image for Diab+BG was duplicated. In Figure 4, Day 12 image for Diab+BG was duplicated. In Figure 6, Day 3 image for Diab, Day 9 image for Diab, Day 12 image for Cont were duplicated and, consequently, Figure 6 legend was incorrect.

“Effect of BG treatment on the expression of CD31 in wounded skin tissues of diabetic mice. CD31 staining of (**A**) Day-3; (**B**) Day-6; (**C**) Day-9; (**D**) Day-12; (**E**) Day-15 wounds; and (**F**) quantitative angiogenesis (400) (n = 6 wounds from 3 animals/time point/each group).”

now reads:

“CD31 staining of (A) Day-3; (B) Day-6; (C) Day-9; (D) Day-12; (E) Day-15 post-wounding; and (F) quantitative angiogenesis expression (400x) and scale bar = 20 µm; (n = 6 wounds from 3 animals/time point each group).”

Finally, in Figure 7, Day 3 image for Cont was duplicated.

The original Figures 1, 4, 6, 7 and their accompanying legends appear below.

**Fig. 1:**
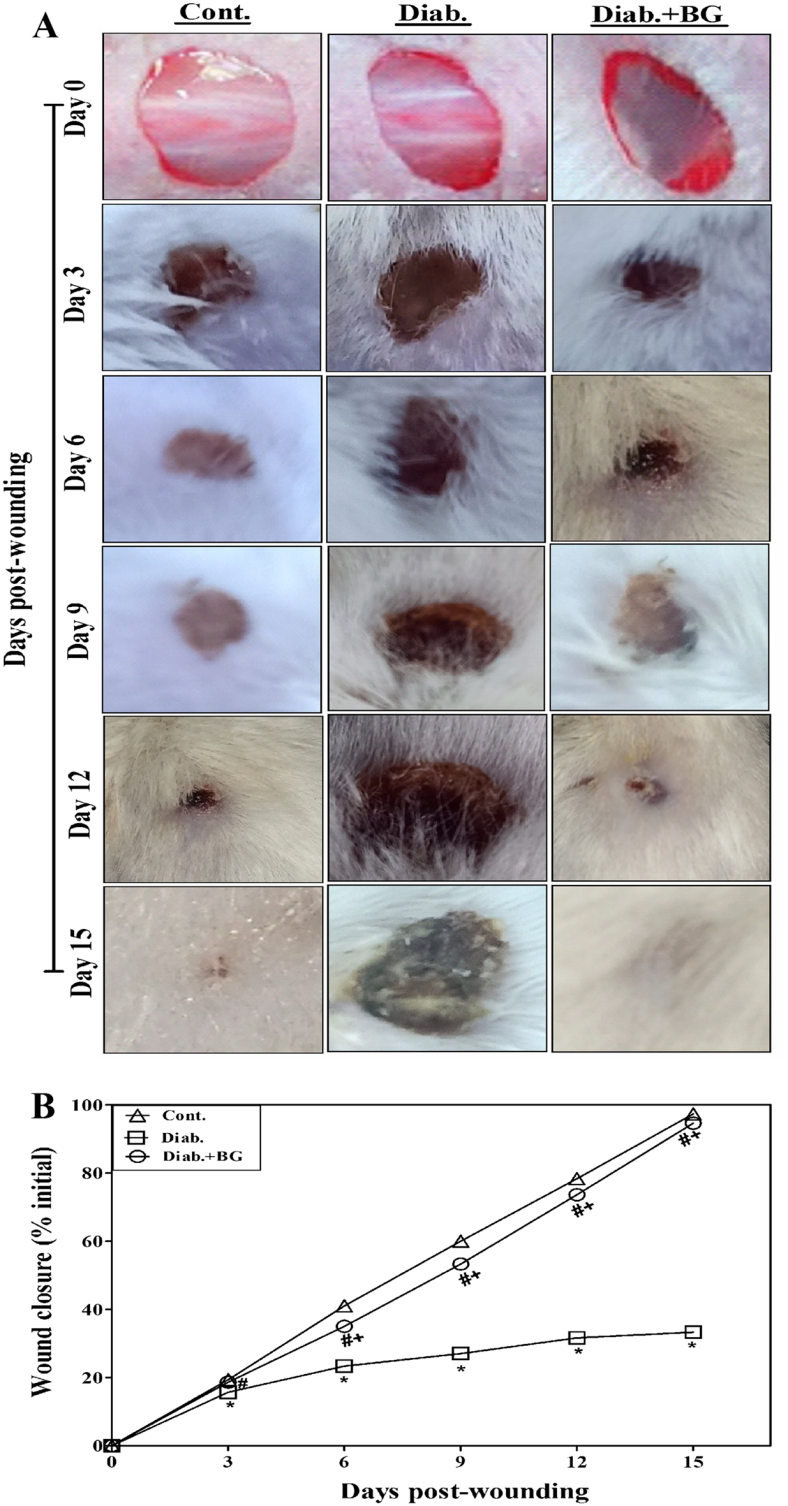
Effect of BG treatment on wound closure. (**A**) The wound areas were photographed at the designated times. The images from day zero were taken instantaneously following the injury. Each group’s illustrative data are drawn from 20 individual mice that are shown. (**B**) The data that was gathered for variations in the percentage of wound closure from 20 individual animals in each group is displayed.

**Fig. 4:**
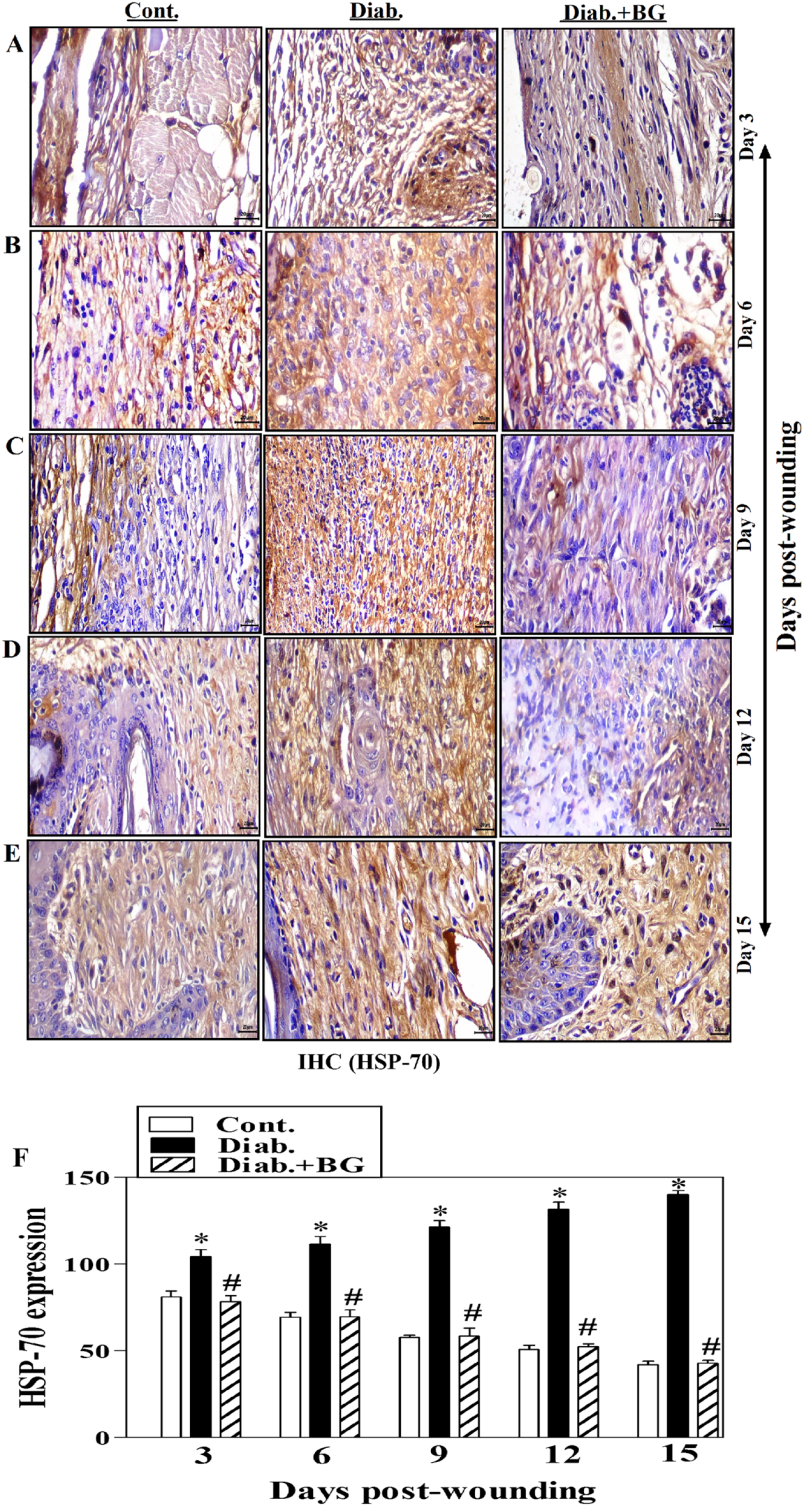
Impact of BG treatment on the expression of HSP-70 in wounded skin tissues diabetic mice. HSP-70 staining of (**A**) Day-3; (**B**) Day-6; (**C**) Day-9; (**D**) Day-12; (**E**) Day-15 wounds; and (**F**) quantification of HSP-70 expression (400$\times$) (n = 6 wounds from 3 animals/time point/each group).

**Fig. 6:**
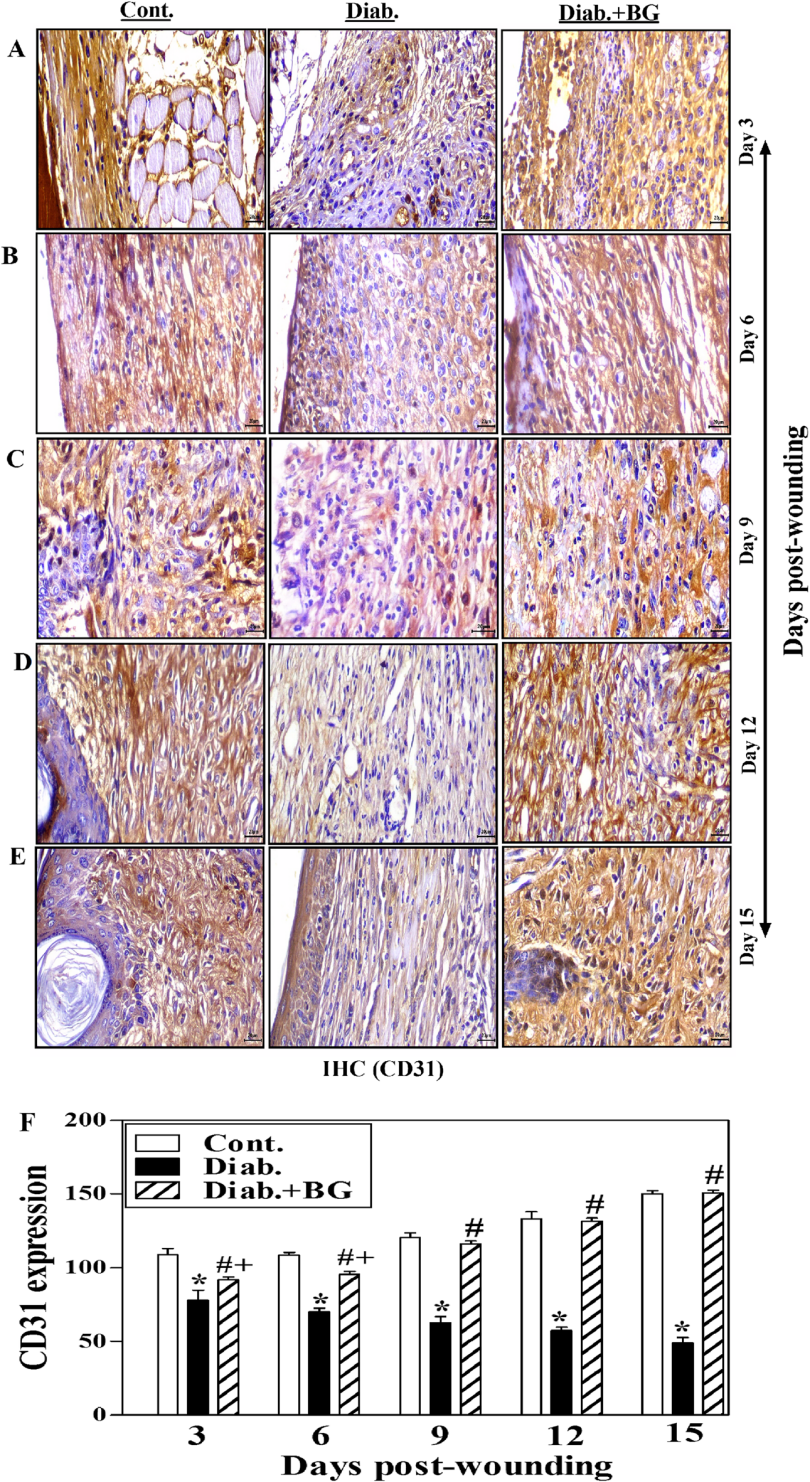
Effect of BG treatment on the expression of CD31 in wounded skin tissues of diabetic mice. CD31 staining of (**A**) Day-3; (**B**) Day-6; (**C**) Day-9; (**D**) Day-12; (**E**) Day-15 wounds; and (**F**) quantitative angiogenesis (400$\times$) (n = 6 wounds from 3 animals/time point/each group).

**Fig. 7:**
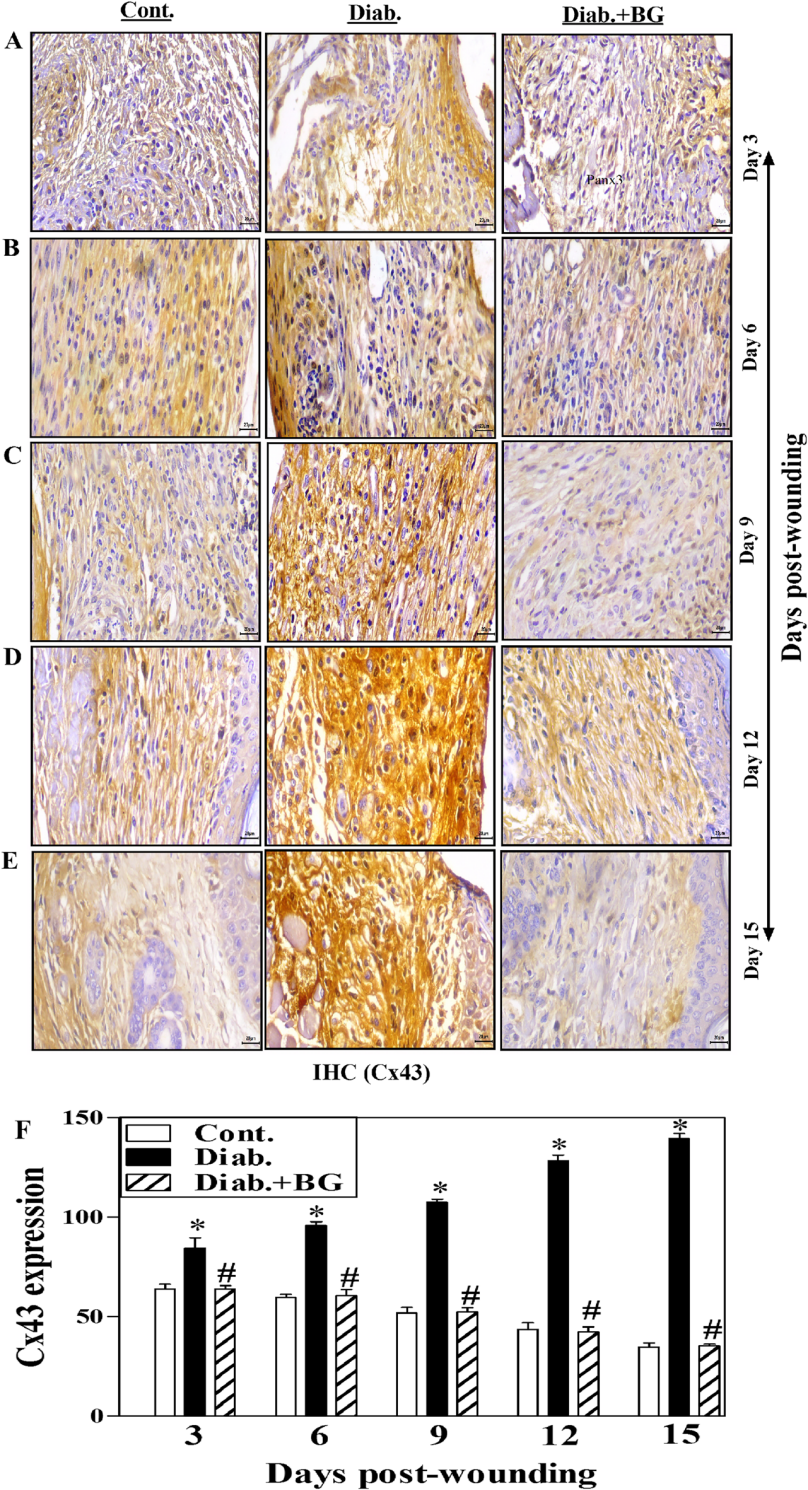
Influence of BG treatment on the expression of Cx43 in wounded skin tissues of diabetic mice. Cx43 staining of (**A**) Day-3; (**B**) Day-6; (**C**) Day-9; (**D**) Day-12; (**E**) Day-15 wounds; and (**F**) quantification of Cx43 expression (400$\times$) (n = 6 wounds from 3 animals/time point/each group).

The original Article has been corrected.

